# Psychosocial Needs and Preferences for Care among Adolescent and Young Adult Cancer Patients (Ages 15–39): A Qualitative Study

**DOI:** 10.3390/cancers14030710

**Published:** 2022-01-29

**Authors:** Viswatej Avutu, Kathleen A. Lynch, Marie E. Barnett, Jacqueline A. Vera, Julia L. Glade Bender, William D. Tap, Thomas M. Atkinson

**Affiliations:** 1 Department of Medicine, Memorial Sloan Kettering Cancer Center, New York, NY 10065, USA; tapw@mskcc.org; 2 School of Global Public Health, New York University, New York, NY 10003, USA; lynchk@mskcc.org; 3 Department of Psychiatry and Behavioral Science, Memorial Sloan Kettering Cancer Center, New York, NY 10022, USA; barnettm@mskcc.org (M.E.B.); veraj@mskcc.org (J.A.V.); atkinsot@mskcc.org (T.M.A.); 4 Department of Pediatrics, Memorial Sloan Kettering Cancer Center, New York, NY 10065, USA; gladebej@mskcc.org

**Keywords:** AYA, qualitative studies, LGBTQ+, psychosocial, survivorship

## Abstract

**Simple Summary:**

Adolescents and young adults (AYAs) are a unique population: they are a diverse group between the ages of 15–39 years with distinct needs and experience numerous developmental milestones during this age range. Notably, AYAs have faced worse outcomes in cancer care, both with shorter survival and worse quality of life compared to children and older adults. Understanding AYAs’ psychosocial, communication and informational needs is crucial to addressing this disparity and improving cancer care delivery. By hearing directly from AYAs, we are able to capture nuances of their experiences and provide clinical recommendations to healthcare providers involved in the care of AYAs with cancer. This study specifically interviewed AYAs with cancer to understand their perspectives, identify needs and to develop recommendations for cancer care delivery and accommodations across the cancer experience.

**Abstract:**

Adolescents and young adults (AYAs) require a multidisciplinary approach to cancer care due to their complex biopsychosocial situations and varied developmental maturity. Currently, age and diagnosis determine referral to pediatric or adult oncology, with differing treatment paradigms and service utilization patterns, contributing to suboptimal improvements in outcomes. Understanding the unique perspectives of AYAs is essential to designing patient-centered AYA services. Thus, we conducted six focus groups with AYAs (*n* = 25) treated by medical or pediatric oncologists to evaluate: (1) the unique experiences of cancer care as an AYA; (2) AYA-specific information needs and communication preferences; and (3) recommendations for service provision, delivery and accommodations for AYAs. Transcripts were analyzed using inductive thematic content analysis and identified six major themes to inform clinically-actionable recommendations and the development of a patient-reported outcome measure: (1) AYAs experience social isolation and loss of independence; (2) AYAs have an uncertain sense of the future and need conversations around survivorship and long-term and late effects; (3) AYAs desire greater control over discussions with their care team; (4) AYAs need additional navigational and social/caregiver supports; (5) AYAs prefer an inclusive AYA space in the hospital; and (6) LGBTQ+ patients experience distinct concerns as AYA cancer patients. These will form the basis for specific and tailored clinical recommendations to improve AYA cancer care delivery.

## 1. Introduction

Adolescents and young adults (AYAs) with cancer are distinct, both physically from a disease pathophysiology perspective and psychologically from a developmental perspective [1]. Defined by the National Cancer Institute (NCI) as individuals between 15–39 years old, this diverse age group experiences a range of physiological changes and social transitions [1,2,3]. Navigating a cancer diagnosis during this stage disrupts nearly all aspects of life [3,4]. In addition, AYAs commonly face worse outcomes in cancer care, both with shorter survival and worse quality of life, compared with children and older adults [5,6]. Currently, age and diagnosis typically determine referral to pediatric or adult oncology, each with differing practices, procedures and service utilization, including psychosocial and supportive services. In general, AYA development is largely recognized as a multidimensional process, with physical, psychological, social and environmental changes. Few studies in psychosocial oncology capture the unique ways cancer treatment interacts with, disrupts and promotes these developmental processes during and after treatment [1,7,8]. This is due in large part to several factors, including: differences in the nature and behavior of AYA cancers; lower participation in clinical trials; varying treatment plans; and inconsistent use of supportive services, such as social work, fertility services, sexual health, physical and occupational therapy, pain and palliative care and rehabilitation medicine [9,10]. Systemically, non-uniform treatment paradigms in conjunction with differences in tumor biology and lower accrual to clinical trials in the AYA population have led to marginal improvements in overall survival over the last three decades compared to therapeutic improvements seen in younger and older patients [3,11,12,13,14,15].

As the holistic psychosocial perspectives and needs of AYAs are understudied and underrepresented, there is a critical need for greater patient-centered research that addresses the unique needs of AYAs, including: psychosocial care; quality of life; treatment adherence; decision-making; cognitive functioning; and emotional well-being [14,15,16,17,18,19,20,21,22,23,24]. Therefore, this qualitative study sought to explore AYAs’ psychosocial and cancer-specific needs that are crucial throughout their cancer experience. The aims were to: (1) explore the unique experiences of cancer care as an AYA, including self-identified physical, social and emotional concerns; (2) identify AYA-specific information needs and communication preferences, including the appropriateness, timing, and depth of information delivered; and (3) provide recommendations and direction for service provision and delivery, and accommodations for AYAs throughout their cancer experience.

## 2. Materials and Methods

### 2.1. Ethical Approval

The focus groups for this study were conducted as an extension of a quality improvement initiative at Memorial Sloan Kettering Cancer Center (MSK; New York, NY, USA) to design and develop a cancer care program for AYAs. For the present analysis, we sought a retrospective waiver from the hospital’s Institutional Review Board to identify generalizable themes to inform clinical practice recommendations [Protocol 21-011]. All participants and their legal guardians when appropriate gave informed verbal consent at the start of the group, following a discussion of the group’s purpose, potential risks and benefits, and steps to protect confidentiality were undertaken throughout focus group conduct and analysis.

### 2.2. Participant Recruitment

Participants were eligible if they were between the ages of 15 and 39, treated in the medical or pediatric oncology service and English-speaking. Providers (oncologists, nurse practitioners, psychologists and social workers) were asked to identify and refer a list of eligible patients to the study team. Referred patients were then contacted by a study coordinator who confirmed eligibility and assessed interest in participating in a 90-min focus group. The coordinator informed potential participants of the purpose of the focus group and emphasized that the study was voluntary and would have no bearing on their clinical care. Interested patients were then emailed details regarding group timing and location.

### 2.3. Data Collection

We conducted six focus groups, stratified by sub-group: (1) participants ≥18 years treated in medical services; (2) participants ≥18 years treated in pediatric services; (3) a mixed group of participants ≥18 years treated by either medical or pediatric service; (4) adolescent participants aged 15–17 years; (5) participants who identify as LGBTQ+ (Lesbian, Gay, Bisexual, Transgender and Queer or Questioning). This allowed for the opportunity to explore the unique needs of each patient group in-depth, as well as observe the interactions between AYAs referred into different treatment services. Focus groups were conducted until thematic saturation regarding common psychosocial experiences and needs was achieved across the dataset (i.e., “meaning saturation”) while stratification into heterogeneous groups allowed for the identification of issues unique to each particular group (i.e., “code saturation”) [25]. The study team (KAL, AV, MB, JV) iteratively reviewed focus group notes and transcripts throughout the data collection period to identify whether meaning saturation had been achieved. While we initially did not stratify our groups aside from their primary oncology service (i.e., medical or pediatric), initial data analysis suggested that we were not achieving thematic saturation for specific groups–namely those younger than 18 years and those who identified as part of the LGBTQ+ community. Thus, to better understand how the experiences of these subgroups was different from the larger AYA group, we chose to recruit specifically from individuals belonging to these subgroups for subsequent focus groups.

The first two groups were conducted in-person, and the next three were held over a secure videoconference platform (WebEx, Zoom) for participant convenience and to adhere to social distancing guidelines in response to the COVID-19 pandemic. Recent qualitative research has demonstrated that virtual groups elicit comparable thematic content to in-person groups, are acceptable to many participants and can increase participant diversity [26,27,28,29]. A qualitative methodologist with expertise in focus group moderation (KAL) led each discussion using a semi-structured guide (Supplementary Materials Table S1), with one additional investigator in each group taking notes focusing on non-verbal cues or observations (VA/MB). The discussion included topics such as: experiences of care as an AYA; self-identified physical, social and emotional concerns, including concerns that they feel are suboptimally addressed; information needs, including the appropriateness, timing, and depth of information delivered. Probing questions were also generated during the session, allowing the interviewer to reflexively respond to relevant topics as they emerged during the discussion. Following the focus group, participants completed a brief questionnaire assessing sociodemographics. Interviews were audio-recorded and transcribed verbatim.

### 2.4. Analysis

Demographic data were analyzed descriptively. Focus group transcripts were analyzed using an inductive thematic content analysis approach [30,31]. Transcripts were first coded independently by each member of the study team (KAL, AV, MB, JV) using open coding. The study team first created initial descriptive and interpretive codes and decision rules for applying each code. After establishing a codebook, the team coded the subsequent transcripts, meeting regularly to achieve consensus on emerging patterns, refine code definitions and resolve discrepancies in coding. Then, the study team grouped similar codes into categories–thematic labels supported by the text. A secondary analysis of code categories was then completed by each member of the above study team, which involved independently reviewing the coded content to identify key findings. Then, the team met as a group to reach consensus on the instances of thematic saturation, resolve discrepancies and identify primary themes and subthemes. Qualitative software NVivo Pro 12.0 facilitated the analysis [32].

## 3. Results

### 3.1. Demographics

A total of 25 individuals participated in focus groups, with 2–7 participants per group. The average age was 26.8 years (range 16–39 years), with relatively even gender representation (54.2% identifying as female) and 70.8% identified as Caucasian, consistent with institutional patient demographics. Education and household income were well distributed. Nine participants were treated in the medicine service, 13 in the pediatric service and two were treated in both services. Notably, one participant was a caregiver to a patient in the group. Full demographics are listed in Table 1.

### 3.2. Major Themes

We grouped our codes into nine major categories (desire for social relationships, informational needs, physical space preferences, communication experiences, post-treatment and survivorship concerns, psychosocial concerns, disease-related concerns, suggestions for AYA-specific programming and LGBTQ+ nuances). From a review of these categories, we identified six major themes and one sub-theme, as listed below. Table 2 includes a breakdown of major codes, categories and themes. Table 3 includes theme descriptions and supporting participant quotes.

**Theme** **1.** *Social isolation and loss of independence.*

Across groups, participants described their cancer experience as profoundly isolating. As one participant stated,

*“So I had cancer since my senior year of high school and between college, and definitely the, definitely one of the hardest parts was all my friends going away to school and me not being able to go to school just yet because I’m still recovering. And it definitely gets really lonely sometimes, you know.”*

(18+, treated in Pediatric Service Group)

Many noted that their peers were unsure how to interact with them, and it became difficult to navigate social activities. As one participant described,

*“I’m in chemotherapy, so I feel not great a lot of the time and seeing friends in person is something that has changed a lot. Being spontaneous is a lot more difficult.”*

(18+, Treated in Medical Service Group)

This isolation was compounded by a perceived loss of independence; participants discussed their frustration at needing to rely on their caregivers, who were often parents, feeling that they were “giving up” the newfound freedoms and emerging self-reliance of young adulthood. Some felt that their life had been “put on hold” due to cancer:

*“I was living away from home, working when I was diagnosed. When I was diagnosed I relocated back home and was living with my parents then after being away for six years. And I felt like it was challenging. My parents were great and they attended all of my meetings and came to the hospital with me every day, but it was difficult going from being independent to then depending on my parents again.”*

(18+, treated in Pediatric Service Group)

This sense of isolation extended to the cancer treatment setting as well. Participants said that they were either the oldest person in the pediatric clinic or the youngest person in the medical clinic:

*“I had a friend that when I was diagnosed told me that she had gone through leukemia when she was in her twenties and she was like I literally had no idea that other people my age could be diagnosed with cancer because her experience was so isolated. She was the only person her age in the adult ward. She was like, ‘You’re the only other person I’ve ever met who is my age, who has gone through something like this.’”*

(18+, treated in either Service Group)

Participants also noted that they were sometimes mistaken for a caregiver or family member while waiting for appointments.

***Theme*** ***1.1.*** *Desire to connect with peers.*

Due to this isolation, participants expressed interest in meeting other AYAs diagnosed with cancer, during and post-treatment. As one participant described,

*“A lot of times when you go to treatments or appointments you’re the only young person there. Everyone’s older, so you kind of—feel like an outsider I guess. It’s nice to connect with other people going through the same thing.”*

(18+, treated in Medical Service Group)

However, many participants found it difficult to initiate conversations with other AYAs they encountered in the medical setting. For example:

*“I think just being in Ped[iatrics] you like you do this visual clock of anyone who is over the age of 15 when you’re there […] but because you’re getting a blood transfusion or you’re like knocked out you have no way of sort of saying, ‘OK, I’ve caught this person and now I’m going to go talk to them and introduce myself,’ and be like, ‘Yo, what kind do you have?’ that facilitation, just feels like next to impossible and you never know how the other person is going to take it. I always felt scared.”*

(18+, treated in either Service Group)

Across groups, participants suggested a “warm handoff” approach, where members of the care team could suggest patients to connect with. For example,

*“It would be helpful for someone there to facilitate the introduction between maybe two young adults they might be like, hey, there’s another twenty-something year old. Would you want to go introduce yourself or talk to them?”*

(18+, treated in either Service Group)

Importantly, AYAs expressed that a warm handoff should only present the opportunity to connect, rather than a mandatory interaction, leaving patients the autonomy to decide how, where and when to engage with their peers. In the LGBTQ+ groups, participants felt it could be especially helpful to have the option to meet LGBTQ+ allies in their medical center, including patients and providers. For example:

*[Speaker 1]: “…if someone had come to me and I am like hey there’s a group of like queer cancer patients at [the hospital] and we are meeting up at 14th floor lounge at this time, I would be maybe interested […] if I knew that it was a safe space then like maybe I would have done that, and it was also a way to crop the divide between peds and adults units, right? Like I met people in New York now who were like oh I was at [this hospital] at the same time but we were in different units, so I never saw them, I don’t know. Maybe queerness is a way to connect the two.*

*[Speaker 2]: Ya this is speaker 2. I would have checked that out if I was well enough.”*

(LGBTQ+ Group 2)

**Theme** **2.** *Uncertain sense of the future and a need for conversations around survivorship, long-term and late effects.*

AYAs expressed an uncertain sense of the future and what to expect post-treatment. During treatment, participants were unsure of survivorship and what it entailed for someone their own age. As one participant described,

*“Something that I really struggled with when I was in treatment was believing that there were people [my age] that had survived, because I just wasn’t seeing them. Being inpatient, you’re only seeing the people who are currently in treatment and then the people who aren’t in treatment aren’t at the hospital anymore, right? You just don’t have access to them in any way […] and so you only have the narrative of people your age who are dying or people your age who are in treatment and the concept of survivorship for me was just like so vague, and I didn’t know how to access it, and I just didn’t believe that it was possible.”*

(18+, treated in either Service Group)

In addition, participants expressed lacking knowledge about long-term treatment and late effects. This was especially the case among those treated in the medical service, who were often decades younger than other patients. For instance, one participant stated that they desired more information,

*“Especially with issues like after surgery in terms of mobility when there are patients who have similar issues or similar diseases even in their 70s. What does mobility look like after that versus [even] five or ten years younger is very different.”*

(18+, treated in Medical Service Group)

Finally, AYAs desired more open discussion with their care team on the transition to survivorship life and psychosocial expectations. With active treatment taking place during formative years of their young adulthood, many participants felt unsure about how to navigate their social and professional life post-treatment, especially as many had to pause or postpone major milestones in their personal and professional lives:

*“I think I agree with the point about having had more communication about survivorship. I kind of felt like I treated for ten months and I was fine for ten months and then all of a sudden it was ‘You’re good to go, you can go back to work now.’ And it was really like what does that mean? Like can I move? Can I relocate? Can I go back to work full time, part time? What does that look like?”*

(18+, treated in Pediatric Service Group)

**Theme** **3.** *Greater control over discussions with the care team.*

In each focus group, AYAs desired more control when communicating with their care team. This was particularly the case for individuals diagnosed during adolescence, who described persistent parental involvement in treatment discussions even as they themselves matured:

*“My parents had to be at all my appointments and one thing that I hated was that my doctors wouldn’t talk to me, and they would talk to them because they would be like adults in the room, but like doctors, like I feel like they filter information and then when they give it to my parents then that would get filtered again. So it was like by the time I got the information—what I was missing. And then when I came here, like it was just me. So it was great. My doctors talked to me and I can ask questions, and then I remember one time recently I brought my mom for my appointments and the same thing happened even though I’m 20 now, it’s like, am I in the room or is my mom? So, I definitely like being one-on-one with doctors.”*

(18+, treated in either Service Group)

For certain topics, such sexual health or fertility, participants voiced a need to control how, where and with whom the content is delivered. For example, the presence of loved ones during sensitive discussions can impede or facilitate communication, as one participant described:

*“I think that the most difficult conversations that I had with my parents in the room were conversations about fertility. They were things that I hadn’t quite thought of that doctors were asking me what I wanted to do. And I hadn’t gotten a chance to talk about it with my fiancé yet, and I didn’t mind my parents being in the room, but it made it a little bit more challenging having an extra voice giving input in the conversation.”*

(18+, treated in Pediatric Service Group)

In most groups, participants discussed the challenge of conversations surrounding fertility. In addition to dealing with influences from family, they wished they had received information earlier in their treatment or had more time to process their options. As one participant shared:

*“I wish in a big way that my fertility had been talked about differently. I, I am going to cry when I talk about this. When I like asked about my fertility, you know I was 25, they said that I did not have time to pursue any fertility preservation and as anecdotal evidence for like that I would be fine, the doctor pulled a picture of a woman who had like survived osteosarcoma treatment and had many many children, and I just feel so much rage about that in my body now because no, that’s not going to be the case for me.”*

(LGBTQ+ Group 1)

In general, participants favored direct communication with their medical team regarding realistic expectations for treatment and side effects. However, some participants noted that too much information can be overwhelming and they desired a way to indicate a preference for the amount of information to receive at a given time. For example,

*“One of my doctors when I was filling out the forms, was like ‘What kind of patient are you? do you want a lot of details? Do you not want any detail? Do you want us to tell you when someone’s there, or if someone’s with you, or do you want us to wait for them to leave?’ And I was like, this is awesome. I haven’t seen this on any other paperwork and I loved it.”*

(18+, treated in either Service Group)

**Theme** **4.** *Need for additional navigational and social/caregiver supports.*

While some AYAs discussed their experiences living with family during treatment, others described the opposite experience of relocating for a clinical trial. AYAs expressed the need for additional resources to help navigate treatment and daily life in a new environment. For example,

*“I had to learn the ins and outs of public transportation and I had to find a place to live, and then I had to move two or three months after I moved there, and so it was pretty hectic […] I’ve never been here before, so maybe [I could have used] like a packet of ‘this is how the subway works.’”*

(18+, treated in Medical Service Group)

Rather than living in a state of arrested development, participants faced an accelerated, and at times forced, transition to adulthood in an unfamiliar setting. They noted specific challenges of dealing with a cancer diagnosis during a “transitional” period in their lives. As one participant described,

*“I think that people that tend to be beyond this age group, or before this age group, would have parents or spouses […] probably in the picture in most cases, and I think one thing that you have to address immediately, if you’re doing chemotherapy right off the bat, is who is going to accompany you to your appointments?”*

(18+, treated in Medical Service Group)

For AYAs, coordinating various aspects of their cancer care is complex as they balance their innate autonomy and the biases of their support system. For example, decisions regarding navigating changes to healthcare proxies are multifaceted, as described by one of our LGBTQ+ participants,

*“So one my providers were used to deferring to the mother and not recognizing that the person who lived with me and built a life with me was probably a little bit more attuned to how I was behaving, even though my mother had been my primary caregiver the past […] nobody had talked to me before I started treatment again about updating my proxy, even though they knew very well that my social situation had changed and that my partner would be my primary caregiver. They didn’t think to tell me to update my proxy and so I didn’t.”*

(LGBTQ+ Group 2)

Therefore, in addition to the stressors of treatment, AYAs often need to navigate a complex legal and sensitive family environment, as well as contend with providers who may not fully appreciate the vital role of their partners or other support members in their care. Other participants added that partners or close friends may not necessarily label themselves as “caregivers.” This made it especially difficult for these individuals to obtain access and resources needed to support themselves and the patient.

*“It was kind of a gray area and we were so young, I think it would be presumptuous calling herself a caregiver but though looking back on it, she absolutely was. Like she was there at home, like she was on call if something went wrong, visited us every day, you know. I think anyone paying attention would have realized that hey you are a caregiver. Here’s some support if you want it.”*

(LGBTQ+ Group 2)

**Theme** **5.** *Developing an inclusive AYA space in the hospital.*

In each group, participants commented on the physical characteristics of the clinic, providing suggestions for fostering an inclusive, AYA-friendly environment. Participants treated in the medical service noted the potential “triggering” experience of being the youngest person in the waiting room:

*“I think the triggering part for me was that you look around I was the youngest person in the room. So that was really hard to kind of just look around and be like how am I this young and have this disease but I mean it’s sad to see older women going through the same thing. So I am the complete opposite spectrum, nobody was crying but like that’s the silence that kind of resonated in the waiting room over them speaking loudly on their cellphones. But like other than that the triggering part of it all is that you look around and you are like I am the youngest person here!”*

(LGBTQ+ Group 1)

In general, the waiting room in the medical service was described as “crowded” and “cold.” In contrast, participants treated through the pediatric service generally felt positive about their environment, describing it as “cheerful” and “welcoming.” However, some participants noted that the combination of families and small children moving throughout the space could become “overwhelming:”

*“I know that they like try so hard, I like appreciate all of that staff that work upfront so much, they try so hard. But like that space makes my, like I can’t even describe what it does to me […] part of what happened for me is that I had significant hearing loss from my chemo and so like the particular feeling of like being in that space for like felt pretty cacophonous. I feel like the sound really moves. You know and there can be kids crying or like people having various levels of conversations like the whole thing is happening in one part of it, the amount of sound in that space for me is very overwhelming.”*

(LGBTQ+ Group 1)

Participants expressed a desire for an AYA-specific space that retained the “cheerful” elements of pediatrics while providing a relaxed environment to facilitate interaction with other patients their age:

*“And I’d utilize the lounge if everyone else who were getting treatment were my age too and take a break from the screaming babies, and all the strollers, and everyone and their grandmothers coming to see the kids sort of thing.”*

(18+, treated in either Service Group)

LGBTQ+-identifying participants emphasized the importance of a physical and environmental space that is intentionally inclusive, affirming that all patients belong:

*“You don’t want to be like ‘oh my god that person might not take good care of me because I am queer.’ And like, I don’t think that that was a rational fear at [this hospital] but is definitely a fear that I carry with me in every single medical institution that I go in to. So yeah, I think any kind of representation whether it’s like a rainbow flag or whatever it is, would make it helpful because we sense that, we feel that.”*

(LGBTQ+ Group 2)

**Theme** **6.** *LGBTQ+ AYAs experience distinct concerns as cancer patients.*

AYA patients who identify as LGBTQ+ shared distinct concerns that arose throughout their cancer treatment. Participants in these groups discussed the dual burden of navigating their identity as a young person facing a cancer diagnosis as well as their own gender and sexuality. As one participant described,

*“I think a lot of my experiences are shaped by a lot of adults making decisions about my care and ultimately, like about my body. And how that sort of transitioned to, like growing up in my adolescence, having medical conversations and that gray area of like I can speak for myself, and I sort of can’t speak for myself. And decisions that were being made at a time where I was struggling to sort of figure out a lot of things about my own body and my own sort of, sexuality–just everything. And how medicine and medical care can kind of complicate that.”*

(LGBTQ+ Group 1)

When navigating social relationships, participants felt the burden of belonging to two vulnerable populations–cancer survivors and LGBTQ+ individuals–and the stress of needing “double disclose:”

*“I just feel like at any given moment at any given time I am having to come out to someone in some way. And it’s like roll the dice, is it I have to tell someone I am queer I have to tell someone that I have cancer?”*

(LGBTQ+ Group 1)

During appointments, participants also felt that they constantly had to “come out” to providers. Participants were unsure of the best time to disclose their sexual and/or gender identity, or who on the medical team was aware of a previous disclosure:

*“I think that even if a healthcare provider is not meaning to say something, I would say like my latest conversation about fertility […] like that person is not intending to offend me in any way, but instead of listening to the rest of what she was saying about like possible late effects from my cancer I am now in my head, ‘oh, should I just tell her now or is that important?’ Like will [telling her] help me going forward because like what if I want to have a conversation about fertility that involves a same sex partner? Can I have that conversation with this person? And then my mind is racing on to something else.”*

(LGBTQ+ Group 1)

Participants identifying as LGBTQ+ noted numerous heteronormative assumptions that were made during discussions with their care team, especially during fertility and sexual health discussions, that preempted further individualized conversation:

*“It was assumed in the conversation that do I have a boyfriend [and] the two of us can decide together what we want to do, which is like not necessarily an awful thing to say to a person, but it sort of just like closes it [the conversation] off.”*

(LGBTQ+ Group 1)

Another participant added,

*“In retrospect it might have been good if somebody who was like knowledgeable and educated about how to talk to queer person about that, maybe in retrospect would have liked to have that conversation.”*

(LGBTQ+ Group 2)

Overall, participants in these groups expressed the importance of explicit inclusion and flexibility from the care team. As one participant summarized,

*“My impression of people who work in the medical profession that there is a lot of like black and white thinking. And I think that understanding and serving the LGBTQ+ community requires certain level of flexibility. And understanding that identities change and that’s like a huge part of being LGBTQ+ community. It’s not like, they are not stagnant identities in a way that I think a lot of people would be more comfortable with.”*

(LGBTQ+ Group 2)

## 4. Discussion

AYA patients with cancer are a distinct, vulnerable and underserved population in our current cancer care system [33,34]. As care is often siloed between care delivery systems with a pediatric- or medical-centric focus, the care AYAs receive is often dependent on the model they encounter, an arbitrary process dependent on any number of singular variables (e.g., age, cancer diagnosis, institutional referral patterns, etc.) [11]. Instead, the care of AYAs during cancer treatment and beyond should be guided by a multidisciplinary approach to treatment with an understanding of an individual’s biopsychosocial development [35]. Given the inherent complexity of an AYA’s developmental stage, this study focused on a more detailed understanding of the nuanced variables contributing to these situations and one’s lived experience. These focus groups build on prior literature and identify novel themes related to critical domains in the care of AYA cancer patients: social relationships and independence; uncertainty regarding the future; communication preferences; navigational and social needs and supports; and LGBTQ+ well-being and healthcare delivery. It also illuminates the importance of inclusivity in cancer care and its environment. While other studies have focused on only psychosocial or communication experiences, the themes identified in this analysis reflect a spectrum of unique concerns and clinically actionable recommendations.

While attention to these themes should be part of the standard of care approach to all AYAs, such a biopsychosocial approach is not routinely or systematically provided, often due to a lack of expertise or resources, both financial and non-financial. Thus, these themes both reflect and amplify AYA concerns and priorities and synthesize practical and effective ways to improve the outcomes of AYAs with cancer, at an institutional and system level.

An overarching motif is the burgeoning autonomy and desire for independence of AYAs, which has implications and recommendations for AYA patients, caregivers, providers and researchers. AYAs desire to have control over their cancer care—they wish to have access to all relevant care resources on their terms, but also the ability to shift and adjust their preferences, concerns or priorities [17,36]. They prefer to independently decide on whether, how and when to utilize any given resource [37]. Although they may accept, and even solicit, advice from experts and their social support systems, the ultimate decision must be left to them. This insight has valuable clinical implications for providers: utilizing both repeated standardized and open-ended questions is critical in fostering health literacy and a cancer care system that allows an AYA’s ongoing maturation throughout their cancer journey. 

The cancer experience for AYAs is a particularly socially isolating experience [38]. The relationships that AYAs nurtured prior to their diagnosis invariably change, including the quantity, quality and type of relationships. This is due to modifications in many routine activities in an AYA’s life and a loss of independence in having to forego certain life experiences [1]. Many report a regression to caregiver reliance and place developmental milestones on hold. To blunt the effect of these changes, many AYAs seek in-person and virtual connections with their peers undergoing their own individual cancer experience who can best empathize with their losses. Expectedly, AYAs prefer these connections be made available to them but not mandated or overly structured. However, they still prefer that the cancer care team help arrange and navigate these connections, highlighting the personal trust placed on their care providers. 

While life during active treatment is often regimented and focused solely on medical care, life post-treatment is often unpredictable. This leads to significant anxiety and discomfort for many AYAs, who describe feeling abandoned or losing the consistency provided by the healthcare system that permeated every aspect of their daily life during active treatment [39]. This untethering from the medical system is particularly evident in their experience of survivorship care and the uncertainty regarding potential long-term and late effects of cancer and/or treatment. Addressing these concerns earlier and throughout active treatment can better prepare AYAs for the future and navigation emotional uncertainty. Awareness of these possibilities allows them to mentally prepare for the chance that they may personally experience these sequelae and encourage them to be more proactive in connecting with their care team. Acknowledging that these conversations can be overwhelming, providers must engage with AYAs on these topics as they are ready and capable of doing so.

Patient outcomes may be influenced by an individual’s perception of successful communication of health information, and as found in this study, accommodating an individual and their support system’s communication preferences is vital to an AYA’s ability to process their cancer experience [40]. Communicating health information is particularly dynamic for AYAs, whose support systems may change and adapt to their developmental state (e.g., spousal vs. parental involvement and delegation of caregiver roles) [41]. Additionally, an AYA’s communication preferences often evolve, including how, when and where they prefer information delivered to them. Control over the discussion of sensitive topics (e.g., sexual health, fertility) is especially relevant for AYAs. Directly asking the AYA as to who should be involved and the most appropriate timing of these discussions empowers the AYA and builds confidence and rapport with their care team. This in turn fosters health literacy, leading to a more open and healthy relationship with the healthcare system and acquisition of critical self-advocacy skills. 

Navigating healthcare proxy and caregiver discussions can be complicated for this age group. Recommended communication and care accommodations include allowing revision of who a healthcare proxy is during treatment or survivorship, allowing opportunities to update new relationships or circumstances with cultural sensitivity, especially for patients who identify as LGBTQ+. 

Additionally, these focus groups elicited unique themes at the intersection of AYA and LGBTQ+ concerns and priorities, for which sparse literature exists [42]. Psychosocially, navigating cancer treatment while simultaneously developing one’s gender identity and sexuality can be overwhelming. Care teams must work towards minimizing heteronormative assumptions in communications, increasing flexibility and understanding of how identities can change over time, and appreciating the emotional burden of “coming out” repeatedly to providers [43,44]. Identified clinical recommendations include a need for standardized and inclusive screening and assessment of LGBTQ+ individuals, providing education on inclusive language and communication to providers and creating a safe and inclusive space for LGBTQ+ AYAs [45]. Indication of LGBTQ+ affirmation and allyship is important for LGBTQ+ AYAs as this can positively impact their psychosocial health and cancer experience.

Beyond communication and healthcare relationships, AYAs also identified aspects of hospital environments that should be considered and modified to foster a more inclusive atmosphere. This includes creating a more age-appropriate culture in the care teams and in the waiting rooms and physical locations for AYAs [46].

As the themes above demonstrate, AYA maturity level, needs and preferences vary. Therefore, specific logistical, navigational and informational assessments and resources are critical in supporting an AYA through their cancer experience. Personalized AYA screening tools to measure stress and distress, capture educational and/or vocational needs, assess financial toxicity and understand support systems can provide avenues to alleviate these additional burdens. Resources and culturally sensitive support structures can then be tailored to meet the needs of the AYA where and when they are needed. Unfortunately, most current systems reactively respond to stressors as the etiology and evolution of these stressors is still largely unknown and unpredictable. By better discerning these variables, we can develop systems to proactively prevent and mitigate added stressors, significantly reducing physical and psychological distress and burden and improving the AYAs’ cancer experience. 

### 4.1. Strengths and Limitations

Strengths of this study include a relatively balanced range of age, gender, income, treatment status and varied length of time since diagnosis and since active treatment. Additionally, we purposefully sampled subgroups of AYAs (e.g., 30-year-olds and AYAs who identify as part of the LGBTQ+ community) as these individuals have been historically understudied in AYA research. However, this study is limited by a primarily Caucasian study sample who were recruited from a single comprehensive cancer center. Future studies are needed to explore these themes among a more ethnically diverse sample of patients treated in community settings. As this study was originally conducted for quality improvement purposes, we were unable to link participant responses to information in their medical record. There may be selection bias in that only AYAs with strongly positive or negative experiences volunteered to participate. Furthermore, one of our focus groups included the significant other/caregiver of one of the participants; while this added invaluable context to the nuances in the discussion of the role of health care proxies and caregivers, it may have influenced the responses of other participants. However, given that our participants in the group did not know each other nor the caregiver, we feel this did not meaningfully impede an open and free conversation. In addition, only individuals with availability, access and capacity to join a 90-min focus group were able to participate, meaning that the study may not have captured the needs of acutely ill AYAs or those with limited resources. Nonetheless, we took steps to ensure rigor throughout the data collection and analysis process, including sampling patients across the AYA age range and medical and pediatric services. Our analysis was further strengthened by a multidisciplinary coding team with training in medical oncology, clinical psychology, public health and medical anthropology, who held regular consensus meetings and conducted quality assurance checks on the coding. Finally, this study captures AYA reflections on their experience at a single point in time; future work will engage in longitudinal data collection to assess how AYAs’ needs and concerns may evolve over time. 

### 4.2. Future Directions

By organizing these themes and directly asking AYAs important questions about their cancer experience, improved and individualized care can be provided that addresses this age group’s developmental complexity. Importantly, this allows the care delivery system to shift from a focus on retroactively responding to care concerns to proactively predicting and mitigating such concerns, both at patient- and disease-specific levels throughout the cancer trajectory. There are limited existing comprehensive AYA-specific patient-reported outcomes measures (PROMs) tracking AYA-specific patient-reported outcomes (PROs), the gold standard for capturing disease-related experiences [47,48,49,50,51,52]. PROMs are an essential assessment tool in cancer clinical trials and routine cancer care that systematically capture and assess symptoms and concerns. The concepts elicited and themes identified in this study will inform and guide specific domains and questions to be included in the development and psychometric validation of an oncology AYA-specific PROM.

## 5. Conclusions

AYAs, due to their inherent heterogeneity and complexity of their biopsychosocial development, are particularly sensitive to the disruptions of cancer treatment and sequelae. To achieve better cancer-specific and health-related quality of life outcomes, a tailored approach to their cancer care is required. However, understanding their nuanced concerns and needs is a prerequisite to designing AYA-centered care models and services. Our qualitative study used focus groups and inductive thematic content analysis to describe six specific themes that AYAs self-identified as critical in their care: (1) AYAs experience social isolation and a loss of independence and thus, seek to connect with their peers who can empathize with their struggles; (2) AYAs have an uncertain sense of the future and a need for conversations around survivorship and long-term effects to better prepare for post-treatment life; (3) AYAs desire greater control over discussions with their care team as they are may be overshadowed by their caregivers; (4) AYAs need additional navigational and social/caregiver supports as cancer disrupts all facets of their personal and professional life; (5) AYAs prefer an inclusive AYA space in the hospital where they can connect and find a temporary home; (6) LGBTQ+ patients experience distinct concerns as AYA cancer patients that must be addressed in order to receive holistic care.

By addressing these concerns and implementing the recommendations provided by this study, AYA cancer care can start to be personalized for each individual patient. Furthermore, these themes can form the foundation for tailoring services to meet the needs of AYAs with cancer and, in the future, direct the development of an AYA-specific PROM. This is a vital step towards documenting, understanding, mitigating, strengthening and tracking biopsychosocial variables that influence outcomes for AYAs with cancer.

## Figures and Tables

**Table 1:** Patient Demographics (*n* = 24) ^1^.

Demographic	*N* (%) ^2^
**Group Composition**	
≥18 years treated in medical services	7 (29.2)
≥18 years treated in pediatric services	5 (20.8)
≥18 years treated by either medical or pediatric service	5 (20.8)
Adolescents aged 15–17 years	2 (8.3)
Patients who self-identify as LGBTQ+	5 (20.8)
**Mean age (SD, range)**	26.8 (5.9, 16–39)
**Gender**	
Female	13 (54.2)
Male	10 (41.7)
Genderqueer or Gender non-binary	1 (4.2)
**Race**	
Caucasian or White	17 (70.8)
Black or African American	3 (12.5)
Asian	2 (8.3)
Prefer not to answer	2 (8.3)
**Hispanic, Latinx, or Spanish origin**	
Yes	2 (8.3)
No	20 (83.3)
Prefer not to answer	2 (8.3)
**Current relationship status**	
Single	13 (54.2)
Committed Relationship	7 (29.2)
Engaged	2 (8.3)
Married	2 (8.3)
**Education**	
Some High School	2 (8.3)
High School Diploma	2 (8.3)
Some College	3 (12.5)
Bachelor’s Degree	12 (50)
Master’s Degree	4 (16.7)
Professional Degree	1 (4.2)
**Employment Status**	
Full-time student	6 (25)
Employed, <40 h/wk.	2 (8.3)
Part-time student AND employed <40 h/wk.	1 (4.2)
Employed, >40 h/wk.	8 (33.3)
Full-time student AND employed >40 h/wk.	1 (4.2)
Not employed, looking for work	3 (12.5)
Disabled, not able to work	3 (12.5)
**Annual Household Income**	
Up to $9999	3 (12.5)
$10,000 to $14,999	3 (12.5)
$15,000 to $19,999	0 (0)
$20,000 to $34,999	2 (8.3)
$35,000 to $49,999	2 (8.3)
$50,000 to $74,999	4 (16.7)
$75,000 to $99,999	2 (8.3)
$100,000 to $199,999	5 (20.8)
$200,000 or more	3 (12.5)
**Cancer Treatment** ^3^	
Chemotherapy	21 (87.5)
Surgery	21 (87.5)
Radiation therapy	11 (45.8)
Clinical trial	8 (33.3)
Other *	1 (4.2)
**Time since first diagnosis**	
Less than 6 months	2 (8.3)
6 months to 1 year	3 (12.5)
1 to 2 years	2 (8.3)
2 to 5 years	9 (37.5)
Greater than 5 years	8 (33.3)
**Duration of care at study site**	
Less than 6 months	2 (8.3)
6 months to 1 year	3 (12.5)
1 to 2 years	6 (25)
2 to 5 years	9 (37.5)
Greater than 5 years	4 (16.7)
**Primary oncology service**	
Medical	9 (37.5)
Pediatrics	13 (54.2)
Both Services	2 (8.3)
**Currently undergoing active treatment or therapy**	
Yes	12 (50)
No	12 (50)

^1^ Does not include caregiver participant (*n* = 1); ^2^ Unless otherwise indicated; ^3^ Participants could select more than one cancer treatment; SD = standard deviation; * Other treatment includes targeted therapy, immunotherapy and other therapies not including clinical trials.

**Table 2:** Coding Hierarchy.

Theme	Categories	Major Codes
Theme 1: Social isolation and loss of independence	Unique concerns as an AYA–psychosocial	Sense of isolation
		Changes to social lifestyle
		Life on Hold
		Life Disrupted
		Confused or mistaken identity
*Theme 1.1*: Desire to connect with peers	Desire to connect	Desire to connect with other AYAs
		Warm handoffs
		Interest in community space
		Peer-to-peer connections
Theme 2: Uncertain sense of the future and a need for conversations around survivorship, long-term, and late effects	Post-treatment and survivorship	Post-treatment concerns
		Survivorship
		Remission difficulties
	Unique concerns as an AYA–disease-related	Long-term concerns
Theme 3: Greater control over discussions with the care team	Communication experiences	Influence of others in room
		Communication challenges
		Desired involvement or control in discussion
		Direct or honest communication
	Information needs and preferences	Information overload
	Unique concerns–disease-related	Fertility concerns
Theme 4: Need for additional navigational and social/caregiver supports	Information needs and preferences	Information needs
		Navigating [the hospital]
		Healthcare proxy
		“Things I wish I knew beforehand”
		Caregiver concerns
Theme 5: Developing an inclusive AYA space in the hospital	Physical space	Experiences with physical setting
		Suggestions for AYA space
		Waiting room–Peds
		Waiting room–Adult
	LGBTQ+ experiences and concerns	Inclusive environment
Theme 6: LGBTQ+ AYAs experience distinct concerns	LGBTQ+ experiences and concerns	Sexuality development and identity
		Disclosures or ‘Coming Out’
		Fertility discussions
		Heteronormative assumption
		Inclusive environment

**Table 3:** Key Themes and Supporting Quotes.

Theme	Representative Quotes
Theme 1: Social isolation and loss of independence	*“I probably could have used, I probably could have used the, a friend. Or I just felt very, I felt very alone growing up because I felt somewhere between, and like even like I think more recently I have connected to more programs through MSK […] for like a good stretch in there between like age 16 and 26/27, I was just like floating around like I still have all these problems but like my friends don’t fully get why I am so tired” (LGBTQ+ Group 1)*
*Theme 1.1*: Desire to connect with peers	*“I felt very much like I was seeking some kind of peer support when I was going through treatment and was able to find it just through happenstance because there was another person who was my age and my gender going through a very similar treatment process with a very similar diagnosis and we just happened to be on the same hospital floor. And so if we hadn’t been able to make that connection I think I would have felt incredibly isolated. Even though I was seeing other people that were potentially my age, just like not having a way or a mechanism to reach out or communicate and make that connection was hard.” (18+, treated in Pediatric Service Group)*
Theme 2: Uncertain sense of the future and a need for conversations around survivorship, long-term, and late effects	*“And so the transition from being super present there all the time [in the hospital] and feeling like it’s your second home almost to just having to leave is like, ‘Okay, bye’ You take on a different role and you’re a different person.” (18+, treated in either Service Group)*
	*“I wish that I had asked more questions about all of the different kinds of support that I might be interested in engaging with or receiving after treatment, especially things like financial planning, things like resources, things like even like applying for disability, like any-anything having to do with returning to, to life that I just didn’t talk to my doctors about and I didn’t really attempt to seek those kinds of supports elsewhere. And I wish that I had.” (18+, treated in Pediatric Service Group)*
Theme 3: Greater control over discussions with the care team	*“So now [that I’m] older I kind of feeling like I want to take more my charge of my like care and my parents, my parents obviously are just involved in everything, but I just kind of wish I could do more.” (18+, treated in Pediatric Service Group)*
	*“When I was going to chemotherapy I didn’t have a lot of energy, so I had my mom, or my sister, or my girlfriend take me. After I was done with chemo and whenever I had like an appointment, I basi-cally went by myself. I told my mom when I’d go, but I go in one-on-one with a doctor because I think that’s where I feel most comfortable. I think parents freak out. It’s going to happen. So, I think begin-ning of my stage I had my parents and my sister all with me, but toward the end when I had to go for a checkup when I had to go for like a result after like an MRI or a CT, I just went by myself because I felt more comfortable and I can be 100% honest with the doctor.” (18+, Treated in Medical Service Group)*
Theme 4: Need for additional navigational and social/caregiver supports	*“If you think about other big things that happen in people’s lives, like childbirth and stuff, there is like a whole book, and there’s all these things […] I just feel like there are things [with cancer treatment] that you could potentially have a portal or some like very general info that would be helpful. Like where would I get food if I’m hungry? If I’m nauseous, what do I do about that? Just the like weird things that people need to know, like where are the bathrooms, and how many people can come with you to an appointment and all that? All the weird FAQs of weird stuff that I think does come up in a time that can be so confusing and so scary and there’s so much fear and nervousness, and there’s so much you’re trying to figure out and digest.” (18+, treated in either Service Group*
Theme 5: Developing an inclusive AYA space in the hospital	*“[This discussion] made me think of like the new lounge that they have upstairs for like teens and young adults, and just like making sure like that’s an inclusive space too because if you are going to have like romcoms include queer romcoms, if you are going to have literature, you are going to have books and stuff, include queer writers, like maybe have like a celebrate those queer writers, queer artists, queer musicians. Like make it known to everyone that it’s like ok to be not straight or gender nonconforming. All of that message goes a long, like a little bit goes a long way in my opinion.” (LGBTQ+ Group 2)*
Theme 6: LGBTQ AYAs experience distinct concerns	*“I didn’t identify as gay, I didn’t identify as a lesbian, I just even, what I identify as a queer now, [but] I didn’t have the language for that at the time and so it was really hard for me to like ask questions about sex, I didn’t know how to say, ‘I am this and so I need help with this.’ […] But often I was approached as a straight woman and so I was starting on an identity I wasn’t even, that I wasn’t.” (LGBTQ+ Group 2)*
	*“I was 22 and I was in my first real relationship with my first relationship with someone of the same sex so it was really definitely scary to me […] I mean it sounds kind of funny now but at that time like how will I be sexually active while I am going through chemo and it was something that I thought a lot about [… I asked] ‘can I have sex?’ or whatever, and it was sort of like the answer was ‘oh my god, yes but always use a condom!’ and I was just like, I can’t press further to really get myself to do it. Ya, and I was assumed straight probably throughout most my conversations with doctors” (LGBTQ+ Group 2)*
	*“So, for me, my treatment was like one full calendar year of like constant eighteen chemos. And so, I felt like I lived at [the hospital] that year and so bringing in the fullness of my identity to it was really important. And I came out, I am queer, cis queer to my palliative care team because they asked if there’s anything to know about you. And that was a really nice way to ask that, I thought and gave me that space to come out […] So, I feel like coming out to my palliative care team was a really positive thing for me.” (LGBTQ+ Group 1)*

## Data Availability

A portion of the data presented in this study are available in the Results section of the manuscript and Table 3; the remainder of the data is not publicly available due to the sensitive nature of the transcripts of the focus groups. Please contact the corresponding author for further details.

## Supplementary Materials

The following supporting information can be downloaded at: https://www.mdpi.com/article/10.3390/cancers14030710/s1, Table S1: Sample Focus Group Questions.

## References

[1] Patlak M., Nass S.J. (2014). Identifying and Addressing the Needs of Adolescents and Young Adults with Cancer: Workshop Summary.

[2] Palmer S., Patterson P., Thompson K. (2014). A national approach to improving adolescent and young adult (AYA) oncology psy-chosocial care: The development of AYA-specific psychosocial assessment and care tools. Palliat. Support. Care.

[3] Coccia P.F., Pappo A.S., Beaupin L., Borges V.F., Borinstein S.C., Chugh R., Dinner S., Folbrecht J., Frazier A.L., Goldsby R. (2018). Adolescent and Young Adult Oncology, Version 2.2018, NCCN Clinical Practice Guidelines in Oncology. J. Natl. Compr. Cancer Netw..

[4] Ferrari A., Barr R.D. (2017). International evolution in AYA oncology: Current status and future expectations. Pediatr. Blood Cancer.

[5] Bleyer A., Choi M., Fuller C.D., Thomas C.R., Wang S.J. (2009). Relative lack of conditional survival improvement in young adults with cancer. Semin. Oncol..

[6] Adolescents and Young Adults (AYAs) with Cancer—National Cancer Institute. https://www.cancer.gov/types/aya.

[7] Bleyer A., O’leary M., Barr R., Ries L. (2006). Cancer Epidemiology in Older Adolescents and Young Adults 15 to 29 Years of Age, Including SEER Incidence and Survival: 1975–2000.

[8] Warner E.L., Kent E.E., Trevino K.M., Parsons H.M., Zebrack B.J., Kirchhoff A.C. (2016). Social well-being among adolescents and young adults with cancer: A systematic review. Cancer.

[9] Noone A.M., Howlader N., Krapcho M.A., Miller D., Brest A., Yu M., Cronin K.A. (2018). SEER Cancer Statistics Review, 1975–2015.

[10] Rae C., Shah N., De Pauw S., Costa A., Barr R.D. (2020). System Performance Indicators for Adolescent and Young Adult Cancer Care and Control: A Scoping Review. J. Adolesc. Young Adult Oncol..

[11] Ferrari A., Thomas D., Franklin A.R., Hayes-Lattin B.M., Mascarin M., van der Graaf W., Albritton K.H. (2010). Starting an Adolescent and Young Adult Program: Some Success Stories and Some Obstacles to Overcome. J. Clin. Oncol..

[12] Trama A., Botta L., Foschi R., Ferrari A., Stiller C., Desandes E., Maule M.M., Merletti F., Gatta G. (2016). Survival of European adolescents and young adults diagnosed with cancer in 2000–07: Population-based data from EUROCARE-5. Lancet Oncol..

[13] Forcina V., Vakeesan B., Paulo C., Mitchell L., Bell J.A., Tam S., Wang K., A Gupta A., Lewin J. (2018). Perceptions and attitudes toward clinical trials in adolescent and young adults with cancer: A systematic review. Adolesc. Health Med. Ther..

[14] Keegan T.H., Ries L.A., Barr R.D., Geiger A.M., Dahlke D.V., Pollock B.H., Bleyer W.A. (2016). National Cancer Institute Next Steps for Adolescent and Young Adult Oncology Epidemiology Working Group Comparison of cancer survival trends in the United States of adolescents and young adults with those in children and older adults. Cancer.

[15] Suh E., Stratton K.L., Leisenring W.M., Nathan P.C., Ford J.S., Freyer D.R., McNeer J.L., Stock W., Stovall M., Krull K.R. (2020). Late mortality and chronic health conditions in long-term survivors of early-adolescent and young adult cancers: A retrospective cohort analysis from the Childhood Cancer Survivor Study. Lancet Oncol..

[16] Love B., Crook B., Thompson C.M., Zaitchik S., Knapp J., LeFebvre L., Jones B., Donovan-Kicken E., Eargle E., Rechis R. (2012). Exploring Psychosocial Support Online: A Content Analysis of Messages in an Adolescent and Young Adult Cancer Community. Cyberpsychology Behav. Soc. Netw..

[17] Lea S., Taylor R.M., Martins A., Fern L., Whelan J.S., Gibson F. (2018). Conceptualizing age-appropriate care for teenagers and young adults with cancer: A qualitative mixed-methods study. Adolesc. Heal. Med. Ther..

[18] Sodergren S.C., Husson O., Robinson J., Rhode G.E., Tomaszewska I.M., Vivat B., Dyar R., Darlington A.-S., EORTC Quality of Life Group (2017). Systematic review of the health-related quality of life issues facing ad-olescents and young adults with cancer. Qual. Life Res..

[19] Sodergren S.C., Husson O., Rohde G.E., Tomaszewska I.M., Vivat B., Yarom N., Griffiths H., Darlington A.-S., On Behalf of the European Organization for Research and Treatment of Cancer Quality of Life Group (2018). A Life Put on Pause: An Exploration of the Health-Related Quality of Life Issues Relevant to Adolescents and Young Adults with Cancer. J. Adolesc. Young Adult Oncol..

[20] Reeve B.B., Hays R.D., Bjorner J.B., Cook K.F., Crane P.K., Teresi J.A., Thissen D., Revicki D.A., Weiss D.J., Hambleton R.K. (2007). Psychometric evaluation and calibration of health-related quality of life item banks: Plans for the Patient-Reported Outcomes Measurement Information System (PROMIS). Med. Care.

[21] Zamanzadeh V., Ghahramanian A., Rassouli M., Abbaszadeh A., Alavi-Majd H., Nikanfar A.R. (2015). Design and implementation content validity study: De-velopment of an instrument for measuring patient-centered communication. J. Caring Sci..

[22] Smith A.W., Keegan T., Hamilton A., Lynch C., Wu X.-C., Schwartz S.M., Kato I., Cress R., Harlan L., AYA HOPE Study Collaborative Group (2019). Understanding care and outcomes in adolescents and young adults with cancer: A review of the AYA HOPE study. Pediatr. Blood Cancer.

[23] Telles C.M. (2021). A scoping review of literature: What has been studied about adolescents and young adults (AYAs) with cancer?. Cancer Treat. Res. Commun..

[24] Robertson E., Wakefield C., Marshall K., Sansom-Daly U. (2015). Strategies to improve adherence to treatment in adolescents and young adults with cancer: A systematic review. Clin. Oncol. Adolesc. Young Adults.

[25] Hennink M.M., Kaiser B., Weber M.B. (2019). What Influences Saturation?. Estimating Sample Sizes in Focus Group Research. Qual. Heal. Res..

[26] Irani E. (2019). The Use of Videoconferencing for Qualitative Interviewing: Opportunities, Challenges, and Considerations. Clin. Nurs. Res..

[27] Woodyatt C.R., Finneran C.A., Stephenson R. (2016). In-Person Versus Online Focus Group Discussions: A Comparative Analysis of Data Quality. Qual. Health Res..

[28] Rupert D.J., Poehlman J.A., Hayes J.J., Ray S.E., Moultrie R.R., Morgan D., Robinson J.D. (2017). Virtual Versus In-Person Focus Groups: Comparison of Costs, Recruitment, and Participant Logistics. J. Med. Internet Res..

[29] Wylie C., Anderson J.N. (2021). Conducting virtual, synchronous focus groups in HIV prevention research among Black sexual minority men. JMIR Public Health Surveill..

[30] Miles M.B., Huberman A.M., Saldaäna J. (2014). Qualitative Data Analysis: A Methods Sourcebook.

[31] Creswell J., Poth C. (2018). Qualitative Inquiry and Research Design: Choosing among Five Traditions.

[32] Bazeley P., Jackson K. (2013). Qualitative Data Analysis with NVivo.

[33] Ferrari A., Stark D., Peccatori F., Fern L., Laurence V., Gaspar N., Bozovic-Spasojevic I., Smith O., De Munter J., Derwich K. (2021). Adolescents and young adults (AYA) with cancer: A position paper from the AYA Working Group of the European Society for Medical Oncology (ESMO) and the European Society for Paediatric Oncology (SIOPE). ESMO Open.

[34] Barr R.D., Ferrari A., Ries L., Whelan J., Bleyer W.A. (2016). Cancer in adolescents and young adults: A narrative review of the current status and a view of the future. JAMA Pediatr..

[35] Osborn M., Johnson R., Thompson K., Anazodo A., Albritton K., Ferrari A., Stark D. (2019). Models of care for adolescent and young adult cancer programs. Pediatr. Blood Cancer.

[36] Department of Health (2012). Liberating the NHS: No Decision about Me, without Me.

[37] Wiener L., Zadeh S., Battles H., Baird K., Ballard E., Osherow J., Pao M. (2012). Allowing Adolescents and Young Adults to Plan Their End-of-Life Care. Pediatrics.

[38] Deckx L., Akker M.V.D., Buntinx F. (2014). Risk factors for loneliness in patients with cancer: A systematic literature review and meta-analysis. Eur. J. Oncol. Nurs..

[39] Lea S., Martins A., Bassett M., Cable M., Doig G., Fern L.A., Morgan S., Soanes L., Smith S., Whelan M. (2018). Issues experienced and support provided to adolescents and young adults at the end of active treatment for cancer: A rapid review of the literature. Eur. J. Cancer Care.

[40] Fern L.A., Taylor R.M., Whelan J., Pearce S., Grew T., Brooman K., Gibson F. (2013). The art of age-appropriate care: Reflecting on a conceptual model of the cancer experience for teenagers and young adults. Cancer Nurs..

[41] White V., Skaczkowski G., Thompson K., Bibby H., Coory M., Pinkerton R., Nicholls W., Orme L.M., Conyers R., Phillips M.B. (2018). Experiences of Care of Adolescents and Young Adults with Cancer in Australia. J. Adolesc. Young Adult Oncol..

[42] Clarke M., Lewin J., Lazarakis S., Thompson K. (2019). Overlooked Minorities: The Intersection of Cancer in Lesbian, Gay, Bisexual, Transgender, and/or Intersex Adolescents and Young Adults. J. Adolesc. Young Adult Oncol..

[43] Boehmer U. (2018). LGBT Populations’ Barriers to Cancer Care. Semin. Oncol. Nurs..

[44] Lisy K., Peters M.D., Schofield P., Jefford M. (2018). Experiences and unmet needs of lesbian, gay, and bisexual people with cancer care: A systematic review and meta-synthesis. Psycho-Oncol..

[45] Rossman K., Salamanca P., Macapagal K. (2017). A qualitative study examining young adults’ experience of disclosure and nondis-closure of LGBTQ identity to health care providers. J. Homosex..

[46] Vindrola-Padros C., Taylor R.M., Lea S., Hooker L., Pearce S., Whelan J., Gibson F. (2016). Mapping Adolescent Cancer Services. Cancer Nurs..

[47] Basch E., Deal A.M., Kris M.G., Scher H.I., Hudis C.A., Sabbatini P., Rogak L., Bennett A.V., Dueck A.C., Atkinson T.M. (2016). Symptom Monitoring With Patient-Reported Outcomes During Routine Cancer Treatment: A Randomized Controlled Trial. J. Clin. Oncol..

[48] Vodicka E., Kim K., Devine E., Gnanasakthy A., Scoggins J., Patrick D. (2015). Inclusion of patient-reported outcome measures in regis-tered clinical trials: Evidence from ClinicalTrials. gov (2007–2013). Contemp. Clin. Trials.

[49] Hinds P.S., Brandon J., Allen C., Hijiya N., Newsome R.R., Kane J.R. (2007). Patient-reported Outcomes in End-of-Life Research in Pediatric Oncology. J. Pediatr. Psychol..

[50] Kingsley C., Patel S. (2017). Patient-reported outcome measures and patient-reported experience measures. Bja Educ..

[51] Varni J.W., Burwinkle T.M., Lane M.M. (2005). Health-related quality of life measurement in pediatric clinical practice: An appraisal and precept for future research and application. Health Qual. Life Outcomes.

[52] Basch E., Abernethy A.P. (2011). Supporting Clinical Practice Decisions With Real-Time Patient-Reported Outcomes. J. Clin. Oncol..

