# Supplementary material for: Psychosocial Needs and Preferences for Care among Adolescent and Young Adult Cancer Patients (Ages 15–39): A Qualitative Study
Source: Cancers (Basel). 2022 Jan 29;14(3):710. doi: 10.3390/cancers14030710 (PMC8833451; doi:10.3390/cancers14030710)
Supplement: Supplementary file 1 [file cancers-14-00710-s001.zip › cancers-1580806-supplementary.pdf]

Supplementary Materials: Psychosocial Needs and Preferences for Care among Adolescent and Young Adult Cancer Patients (Ages 15–39): A Qualitative Study

Viswatej Avutu, Kathleen A. Lynch, Marie E. Barnett, Jacqueline A. Vera, Julia L. Glade Bender, William D. Tap and Thomas M. Atkinson

Table S1. Sample Focus Group Questions.

Topic 1: Physical, Social, Emotional Impacts of Cancer Among AYAs.

First, we would like to get a sense of the ways a cancer diagnosis has impacted your life.

- What aspects of your life do you feel were most impacted by your treatment? (probe: relationships, finances, employment, education)?
- What are some of the ways a cancer diagnosis has affected the relationships in your life? a. (probe: family, partners, friendships)?
- Are there any experiences that you think are unique to young adults with cancer?
- What is something you wish friends and family would understand about your experience?
- Thinking back to when you were first diagnosed, what were some of your immediate concerns
    - What kinds of information were you looking for at that time?
- When you were first diagnosed, were you aware of any resources for young adults?

Topic 2: Communication Experiences.

Thank you for everything you have shared with me so far. I would like to discuss further what it has been like speaking with providers about your concerns and experiences.

- What has been your experience discussing concerns with your physician?
- During the course of your treatment, is there anything that you wish your physician had mentioned, or discussed differently?
- Is there anything that you would like to ask your care team, but haven’t?
- Are there topics that you felt uncomfortable discussing because of who was in the room?

- Who do *you* feel would be the right person to talk with about these topics?
- What kinds of resources would you want about (xx) topic?
- At what point during your treatment would you want to discuss (xx) topic?

- What has been your experience going to appointments at [peds/medicine]? a. What aspects have been positive?
  - What do you wish could be improved?

Are there any other aspects of your experience that you would like the share, that we did not have the chance to discuss?
